# Approach for 3D Cultural Relic Classification Based on a Low-Dimensional Descriptor and Unsupervised Learning

**DOI:** 10.3390/e22111290

**Published:** 2020-11-13

**Authors:** Hongjuan Gao, Guohua Geng, Sheng Zeng

**Affiliations:** 1 School of Information Science & Technology, Northwest University, Xi’an 710127, China; xiaojinguo@stumail.nwu.edu.cn (H.G.); shengzeng@stumail.nwu.edu.cn (S.Z.); 2 Xinhua College, Ningxia University, Yinchuan 750021, China

**Keywords:** heat kernel signature, bag-of-words, cultural relic classification, unsupervised learning algorithm

## Abstract

Computer-aided classification serves as the basis of virtual cultural relic management and display. The majority of the existing cultural relic classification methods require labelling of the samples of the dataset; however, in practical applications, there is often a lack of category labels of samples or an uneven distribution of samples of different categories. To solve this problem, we propose a 3D cultural relic classification method based on a low dimensional descriptor and unsupervised learning. First, the scale-invariant heat kernel signature (Si-HKS) was computed. The heat kernel signature denotes the heat flow of any two vertices across a 3D shape and the heat diffusion propagation is governed by the heat equation. Secondly, the Bag-of-Words (BoW) mechanism was utilized to transform the Si-HKS descriptor into a low-dimensional feature tensor, named a SiHKS-BoW descriptor that is related to entropy. Finally, we applied an unsupervised learning algorithm, called MKDSIF-FCM, to conduct the classification task. A dataset consisting of 3D models from 41 Tang tri-color Hu terracotta Eures was utilized to validate the effectiveness of the proposed method. A series of experiments demonstrated that the SiHKS-BoW descriptor along with the MKDSIF-FCM algorithm showed the best classification accuracy, up to 99.41%, which is a solution for an actual case with the absence of category labels and an uneven distribution of different categories of data. The present work promotes the application of virtual reality in digital projects and enriches the content of digital archaeology.

## 1. Introduction

Cultural relics are the testimony of a country’s historical existence, the crystallization of human wisdom, which renders them highly precious for their historical, artistic, and scientific research value. China, an ancient country with a civilization over 5000 years old, has produced a variety of cultural relics with exquisite technology. 

**Figure 1:**
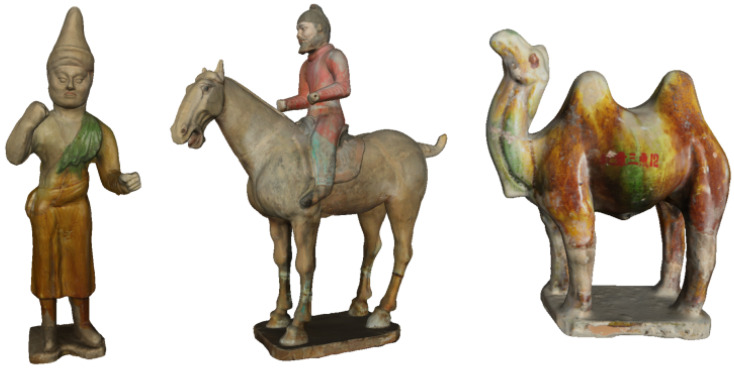
Tang tri-color Hu terracotta sculptures.

In the Tang dynasty, the developed economy and culture produced a prosperous pottery industry. Tri-color Hu terracotta figures, shown in Figure 1, a kind of glazed pottery, embody the unique aesthetic value of the Tang dynasty. This pottery takes magnificence as its modeling, splendor as its color, and warmth as its verve [1]. In many museums, art galleries, and in the hands of private collectors all over the world, there are numerous beautiful and colorful Tang tri-colored crafts that were excavated from tombs and kilns, ranging from 3D ceramic sculptures to various forms.

The non-renewable nature of cultural relics makes them very precious. For enthusiasts or viewers, there are few opportunities to view cultural relics up close, because direct contact with cultural relics is likely to cause damage. If real-world cultural relics were to be digitized into 3D libraries, those interested would be able to query and view high-quality 3D models without direct contact with the real object. With the rapid development of 3D scanning and visualization technologies, heritage virtual management and display platforms have become an important means to store, classify, and retrieve cultural relics, and can also save time for the archaeologist. By obtaining a high-precision 3D model of an artistic relic, researchers can observe more finely the surface morphology and local features. This overcomes the disadvantages of the traditional methods of recording and storing cultural relics and may promote the efficiency of the information management of cultural relics [2]. 

Computer-aided classification and retrieval serve as the basis of virtual cultural relic management. In this stage, cultural relics are classified according to abundant information, such as the existing history and cultures. The anticipated outcome will be a part of the creation of a virtual museum dedicated to integrating cultural relic datasets coming from archaeological excavation activities. In this work, we aimed to develop an effective classification approach for 3D cultural relics that does not require sample labeling in advance. The contributions of this work are as follows: 

Shape descriptors based on heat diffusion have been used in other applications of 3D shape retrieval [3,4], however, they have not been introduced and investigated for the purpose of classifying 3D cultural relics. To reduce the time complexity of calculations and improve the accuracy of feature extraction, we introduced the Bag-of-Words methodology to construct a low-dimensional descriptor called SiHKS-BoW that can accurately represent and describe the features of 3D cultural relics. We proposed an unsupervised learning algorithm to conduct the classification. Our proposed approach can be applied to cases in which the category labels of samples are absent or the distribution of the samples of different categories is unbalanced. 

This paper is organized as follows. In Section 2, the feature extraction method of 3D shapes and some existing cases of cultural relic classification are first introduced. In Section 3, the data processing procedures are described. In Section 4, the details of the proposed approach are given. In Section 5, the experimental results and analyses are presented. Finally, our discussion and conclusion are provided in Section 6 and Section 7.

## 2. Related Work 

### 2.1. 3D Shape Descriptor

In the pattern recognition and computer vision communities, 3D model classification and recognition play an important role in people’s understanding of the physical world. One of the challenges in these tasks is to evaluate the shape descriptor that can accurately capture the topological and geometric information on the surface of a shape [5]. The geometric essence of 3D shape can be succinctly represented by various descriptors extracted from their 3D formats, such as a voxel grid, point cloud, or polygon mesh. At present, 3D shape descriptors can be mainly divided into two categories. Shape descriptors can be considered as a local description of a point of 3D shape, and as a global description of the entire shape [6].

Although many shape descriptors have shown good performance in object recognition and shape matching, they have an underlying condition in that shapes are rigidly transformed, such as spin images [7], local patches [8], curvatures [9], and integral invariants [10]. There are many descriptors that are not sensitive to local geometric variance and are, thus, suitable for isometric transformations. 

Shape descriptors based on heat diffusion [11,12,13,14], were proven to be very effective in capturing the features of 3D shapes. Heat kernel signature (HKS) [11] is a popular local descriptor with the advantage of invariance to isometric deformations and multi-scale properties. Scale-invariant heat kernel signature (Si-HKS) [3] was presented to solve the HKS scale problem with a series of transformations, such as the Logarithmic transformation and Fourier transform. The same research group then introduced the Shape Google approach [14] based on the Si-HKS. 

A global shape descriptor, named a global point signature (GPS) [13], uses eigenvalues and eigenfunctions of the Laplace–Beltrami operator defined on a 3D surface to represent the entire shape. The GPS is invariant under isometric deformations of the 3D shape, yet does not use geodesic distances explicitly. The temperature distribution (TD) descriptor [15], is developed based on the heat mean signature (HMS) at a single scale to represent the entire shape. The L_2_ norm is used to compute the distance between two TD descriptors. Despite the descriptor showing its efficiency and effectiveness, describing the 3D shape of one single scale resulted in an incomplete description, while selecting an appropriate scale is often not simple. 

The effective histogram-based descriptors, such as spin images [7], shape context [16] and color + shape descriptors [17] compressing geometric structures into bins, are more globally discriminate and less sensitive to local geometric variance. The signature of histograms of orientations (SHOT) [18] uses a unique local reference frame to calculate a descriptor along three axes. This descriptor outperforms spin images in object recognition and 3D multi-view reconstruction. 

Using deep learning techniques to extract powerful features [19,20,21,22,23,24] has recently become a popular research area and can be effective for 3D shape classification, retrieval, and matching. Multiple popular representations of the 3D shape have led to the appearance of various deep learning feature extraction approaches. Volumetric Convolutional Neural Networks (CNNs) [21,25] are the pioneers of using 3D convolutional neural networks on voxelized shapes. Multiview Convolutional Neural Networks (CNNs) [26,27] attempt to map the 3D point clouds or shapes into a set of 2D images and, then, apply 2D Conv nets to extract features. Feature-based deep neural networks (DNNs) [28] convert the 3D shape into a vector, define the Eigen-shape and Fisher-shape descriptors as the output of deep neural network (DNN) for learning, and use a fully connected net to classify the shape. In the literature [29], a novel type of deep network named PointNet was designed. The PointNet directly consumes point clouds, and its applications range from object classification, to part segmentation and scene semantic parsing. Other effective and efficient deep network architectures [30,31] were proposed based on PointNet, which achieved promising performance on 3D shape classification tasks. In the literature [32], a deep learning approach to 3D shape classification using spectral graph wavelets and the bag-of-features paradigm was proposed, and a three-step feature description strategy was presented to capture both the local and global geometry of a 3D shape. 

Unlike other traditional 3D shapes features, the features extracted by deep learning are inherent and hidden in the 3D shapes, which provide better overview ability. However, those methods often require a sufficiently large training set, and are not feasible when the training set is small.

### 2.2. 3D Cultural Heritage Classification

In general, the number of samples in a dataset has a large impact on the classifier. Lacking sufficient samples typically results in low efficiency or even overfitting. For cultural relics, the number of samples is relatively scarce. Therefore, some classifiers that require a large number of samples in the database, such as deep learning, are not suitable for cultural heritage classification. Currently, the classification of cultural heritage mostly uses statistical research methods and supervised machine learning methods. 

Different from the classification of other objects, the classification of cultural objects has strong pertinence. It is difficult to compare the classification methods of cultural heritage with any one standard due to their unique characteristics. Computer-aided classification approaches applied to cultural heritage protection and virtual displays can become good examples.

Menze et al. [33] proposed an approach to classify multispectral aster imagery in an archaeological settlement survey in the Near East. The Random Forest method was chosen to classify the set of spectral features. The classifier was trained on the training data set and then applied to the test set. This method can be used in survey planning, the screening of large regions for nature conservation issues, or in landscape archaeology studies. 

Ramil et al. [34] proposed a method based on artificial neural networks (ANN) and laser-induced breakdown spectroscopy (LIBS) to classify archaeological ceramics, including Terra Sigillata. Experimental results showed that the correct classification (higher than 95%) could be achieved objectively and systematically.

Philipp-Foliguet et al. [35] proposed a classification framework of ancient artwork 3D models, and the satisfactory results from this application domain were presented. They deal with database classification based on global and local shape descriptors. An SVM classifier coupled with an active learning strategy was used to retrieve categories of similar objects. 

Hristov et al. [36] presented a classification method of archaeological artifacts that represents ceramic vessels depicted as 2D archaeological drawings. The classification was done by means of a standard k-nearest neighbor (k-NN) algorithm. Experimental results showed that their approach could achieve classification tasks related to the identification of whole vessels and their characteristic fragments. 

Charalambous et al. [37] applied three machine learning methods for the compositional classification of 177 ceramic samples in Cyprus dated to the Early and Middle Bronze Age. The three well-known methods included a standard statistical learning method called k-nearest neighbor, a method based on decision trees, and a complex neural network based on learning vector quantization (LVQ). 

Manferdini et al. [38] proposed a semantic classification method to assist the superintendence of archaeological sites or excavations in the digital management, classification, and visualization of finds inside an advanced repository. 

Desai et al. [39] presented a classification method of archaeological monuments using Content-based Image Retrieval (CBIR) techniques. They applied visual features and the texture of 3D shapes to learn the art form and retrieved similar images from the reference collection. 

Mangone et al. [40] confirmed the highly important role played by different complementary analytical techniques to arrive at a correct archaeological classification of the finds. X-ray diffraction analyses and scanning electron microscopy on ceramic bodies and coatings were performed to identify the provenance of lamps. Multivariate statistical analysis was used to classify various ceramic groups.

## 3. Preliminaries

Mesh and point cloud are two mainstream formats of 3D shape representation. The raw data of cultural relics, obtained using an Artec3D Scanner, are often very large, and there also exists noise data. Therefore, a series of preprocessing of the original data is needed to meet the requirements of the subsequent feature extraction algorithm.

### 3.1. Original Data Pretreatment 

In the process of scanning, the occlusion on object surfaces results in the scanner being unable to collect data in any direction. To avoid incomplete scans, the object must be scanned with multiple angles. The complete 3D model is then obtained by aligning and registering the scanned data onto multiple angles. The roughness of object surfaces may cause a phenomenon of reflection or diffuse reflections when the laser irradiates. The scanners inevitably make errors because of jitter, preheating, and other issues. All these factors result in the formation of holes in the scanning process. The sight interference and occlusion of other objects and many other factors may generate noise points and speckles in the scanned model. Thus, data pretreatment was necessary to correct defects, such as holes, noise points, and speckles. The specific preprocessing steps using Geomagic software are listed below:

(1) Align and register the raw scanned data.
(2) Delete isolated points outside of the object in vitro.
(3) Eliminate noise point and filter speckles.
(4) Patch the holes in the 3D model surfaces.

### 3.2. 3D Mesh Simplification 

In computer graphics, every mesh model is represented as a collection of vertices, edges, and faces. To enhance the performance of applications working with polygonal mesh models, the collection of faces is often reduced to a small subset that holds its basic topology. Table A1 lists the number of vertices and triangle faces of 41 3D models in our dataset before and after simplification.

**Figure 2:**
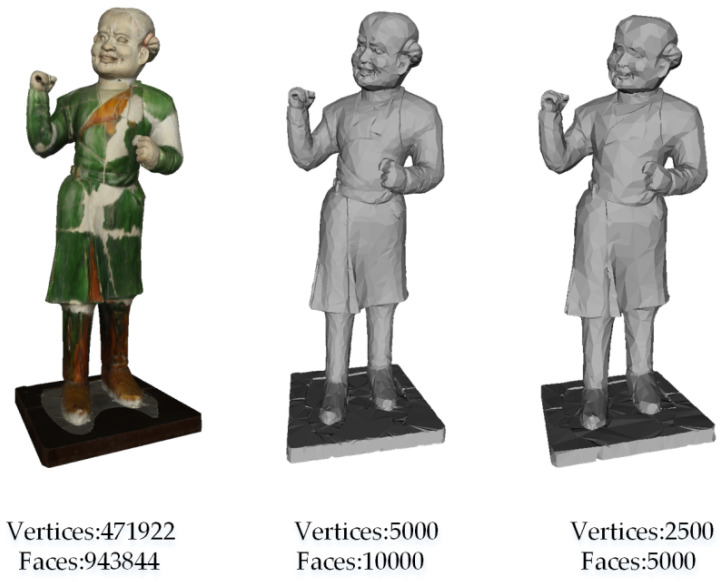
The 3D shape (left) and its simplified versions (middle and right).

Figure 2 is a case in which the triangle faces of model No.86 were reduced from 943,844 to 10,000. As the 3D shape in our dataset is watertight, the number of vertices is reduced, the topology information of the shape is still well preserved and, thus, the ability of feature representation of the 3D model is not weakened after the mesh simplification. 

## 4. The Proposed Method

Our study aimed to develop an effective approach to address cultural relic classification problems. There were three key problems needing to be solved (a) how to obtain a powerful descriptor that well expresses the internal structure of a 3D shape; (b) how to construct a compact low-dimensional feature and make the learning efficient; and (c) how to choose the optimal classifier for our classification task.

**Figure 3:**
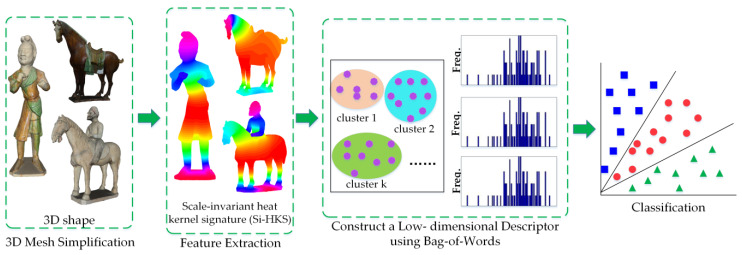
Flow chart of the proposed method.

Figure 3 illustrates each key step of our approach in detail. First, every model was converted to a 3D mesh and simplified. Secondly, the heat kernel value of each point in the corresponding 3D mesh was calculated, and the Si-HKS descriptor was produced. Thirdly, the Bag-of-Words (BoW) methodology was employed to transform the Si-HKS descriptor into a low-dimensional SiHKS-BoW descriptor. Finally, an unsupervised learning algorithm was used to complete the classification task in the 3D cultural relic dataset. 

Why did we choose the Si-HKS descriptor to represent the shape features of 3D cultural relics? First, the heat kernel was linked to the curvature of the 3D shape surface. Points in the flat regions with low curvature tend to dissipate heat while points in the corners with high curvature tend to attract heat. Thus, the heat kernel can characterize the intrinsic geometry structure of a 3D shape well. Second, Si-HKS’s ability to handle data under several distortion scenarios made it ideal for our cultural relic classification task, because there are isometric deformation and scale changes in our 3D models. Finally, the extraction time of heat kernel features is relatively fast, which can meet real-time needs.

Why did we introduce the Bag-of-Words mechanism to conduct a low-dimensional descriptor? There were two main reasons: one was the need to find densities in the feature space, another was the need to standardize the size of descriptor. The heat kernel feature of each 3D model is an N ∗ T dimensional tensor, where N represents the number of vertices of the 3D shape and T represents the time scale or frequency. 3D models typically have tens of thousands of points; thus, the dimensions of the heat kernel descriptor are very high, and the cost of similarity calculation is very high. The Bag-of-words mechanism is capable of feature dimension reduction, which is a common task in the pattern recognition domain. 

The numbers of vertices in each 3D model in our experiments were different after the mesh simplification, which led to inconsistent dimensions in the Si-HKS descriptor. Therefore, we needed to further transform the features into a standard tensor.

Why did we choose the MKDSIF-FCM algorithm as a classifier? Although many supervised learning classifiers fully demonstrated high efficiency in the classification task, a notable problem is that it is not applicable in cases without category labels of the samples. Another situation is that, when the distribution of the samples of different categories is not balanced, the effect of using supervised learning for classification may not be better than unsupervised learning. In a previous study [41], we proposed an improved algorithm of FCM (fuzzy c-means), named MDSIF-FCM. Our experimental results on the public dataset demonstrated its effectiveness, and we obtained good classification accuracy when we applied it to a skull dataset. This is an unsupervised learning method, which was very suitable for the classification task in our current work.

### 4.1. Heat Kernel Feature

Sun et al. [11] proposed a novel point signature called the heat kernel signature (HKS), which is based on the properties of the heat diffusion process on a 3D shape and obtained by restricting the heat kernel to the temporal domain. The HKS is invariant under isometric deformations or perturbations of the objects and demonstrated sufficiently good performance in object recognition and retrieval. In this section, we start with the basics of heat kernel theory. 

#### 4.1.1. Heat Kernel Basics

A 3D shape can be approximately viewed as a Riemannian manifold, possibly without boundaries. The heat kernel denotes the heat flow of any two vertices across a Riemannian manifold M. The heat diffusion propagation on M is governed by the heat equation as follows:

(1) $$\Delta_{M}u(x,t)=-\frac{\partial u(x,t)}{\partial t}$$

where $\Delta_{M}$ and t denote a Laplace–Beltrami operator of M and the diffusion time, respectively. The solution u(x,t)=0 with initial condition ${u(x,0)=u}_{0}\left(x\right)$ describes the amount of heat on the manifold at point x in time t. u(x,t) satisfies the Dirichlet boundary condition u(x,t)=0 for all x∈∂M and all t.

Given an initial heat distribution f:M→ℝ, $H_{t}\left(f\right)$ denotes the heat distribution at all times t, and $\lim_{t\to 0}H_{t}(f)=f$. Here, $H_{t}$ is called the heat operator. It is easy to verify that $\Delta_{M}$ and $H_{t}$ satisfy the relation $H_{t}=e^{-t\Delta_{M}}$ and share the same eigenfunction. If λ is an eigenvalue of $\Delta_{M}$, then $e^{-\lambda t}$ is an eigenvalue of $H_{t}$. 

Heat kernel can be thought of as the amount of heat that is transferred from point x to point y in time t given a unit heat source at x. In other words, $K_{t}(x,.)=H_{t}(\delta_{x})$, where $\delta_{x}$ is the Dirac delta function at ${x:\delta }_{x}(z)=0$ for any z≠x, and $\int {}_{M}\delta_{x}(z)\mathrm{dz}=1$.

The heat kernel on any compact manifold M has the following eigen decomposition:

(2) $$k_{t}\left(x,y\right)=\sum_{i=0}^{\infty }e^{{-\lambda }_{i}t}\varphi_{i}{(x)\varphi }_{i}\left(y\right)$$

where $\lambda_{i}$ and $\varphi_{i}$ are the *i*-th eigenvalue and corresponding eigenfunction of the Laplace–Beltrami operator, respectively. 

#### 4.1.2. Heat Kernel Signatures (HKS)

The heat kernel is restricted to a subset of ${\mathbb{R}}^{+}\times \{x\}$, under mild assumptions, ${{\{k}_{t}\left(x,x)\right\}}_{t>0}$ maintains all the information of ${\left\{k_{t}(x,\cdot )\right\}}_{t>0}$. The heat kernel signature (HKS) describes the heat at a point x on the Riemannian manifold M over the temporal domain t. HKS(x) is defined as a function:

(3) $$\mathrm{HKS}(x):{\mathbb{R}}^{+}\to \mathbb{R},\mathrm{HKS}(x,t)=k_{t}\left(x,x\right)=\sum_{i=0}^{\infty }e^{{-\lambda }_{i}t}\varphi_{i}{}^{2}{\left(x\right)}^{}$$

The value of the HKS is dominated by t and has multi-scale characteristics in the temporal domain. The time parameter t is discretely expressed as t_1_, t_2_, …, t_n_. The heat kernel signature at point x can be regarded as a discrete sequence:

(4) $$k_{t}{(x}_{i}{,x}_{i}{)=(k}_{t1}{(x}_{i}{,x}_{i}{)),k}_{t2}{(x}_{i}{,x}_{i}),\cdots k_{\mathrm{tn}}{(x}_{i}{,x}_{i}))$$

#### 4.1.3. Scale-Invariant Heat Kernel Signatures (Si-HKS)

The HKS is a robust local signature with many good properties, but it is very sensitive to the scale. Given a shape X and its scaled version $M^{\prime }=\beta M$, their eigenvalues and eigenfunctions will satisfy $u^{\prime }=\beta u$, $\varphi^{\prime }=\beta \varphi$ and, therefore, has the equation as follows:

(5) $$h{}^{\prime }(x,t)=\sum_{i=0}^{\infty }e^{{-\lambda }_{i}\beta^{2}t}\varphi_{i}{}^{2}{(x)\beta }^{2}=\beta^{2}{\mathrm{HKS}(x,\beta }^{2}t)$$

Bronstein [3] applied a series of transformations to h for achieving scale invariance. First, the heat kernel signature at point x is sampled logarithmically in time, w.r.t. ${t=\alpha }^{\tau }$ to form the discrete function:

(6) $$h_{\tau }{=h(x,\alpha }^{\tau })$$

Scaling the 3D shape by β will result in amplitude-scaling by $\beta^{2}$ and a time shift by ${s=2\log}_{\alpha }\beta$:

(7) $$h_{\tau }{}^{\prime }{=\beta }^{2}h_{\tau +s}$$

Second, the multiplicative constant $\beta^{2}$ is removed by taking the logarithm of h, and then the discrete derivative to τ:

(8) $${\dot{h}}_{\tau }^{\prime }=\log h_{\tau +1}{-\mathrm{logh}}_{\tau }={\dot{h}}_{\tau +s}.$$

Finally, the discrete-time Fourier transform of ${\dot{h}}_{\tau }^{\prime }$ turns this time shift into a complex phase:

(9) $$K^{\prime }(\omega )=K(\omega ){}^{2\pi \omega }$$

where H and $H{}^{\prime }$ are the Fourier transform of h and $h{}^{\prime }$, respectively, and ω∈[0,2π]. The phase is, in turn, eliminated by taking the Fourier transform modulus (FTM):

(10) $$\left|H{}^{\prime }\;(\omega )\right|=\left|H(\omega )\right|$$

The scale-invariant signature H(ω) at each point x is constructed, denoted as the scale-invariant heat kernel signature (Si-HKS).

**Figure 4:**
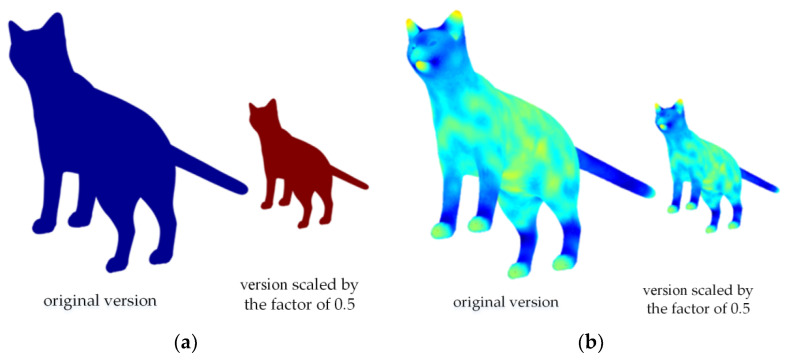
Heat kernel feature computed at a corresponding point on a cat model and its version scaled by a factor of 0.5: (**a**) The heat kernel signatures; (**b**) the scale-invariant heat kernel signatures.

From Figure 4a, the amount of heat remaining on the surfaces of the original model and the scaled model are completely different. This proves that the heat kernel signature is very sensitive to scale changes of the 3D model. Figure 4b shows that the heat distributions of two different versions are virtually identical. Compared with HKS, the Si-HKS algorithm is not sensitive to scale changes. 

### 4.2. Construct a Low-Dimensional Descriptor

The Bag-of-Words (BoW) mechanism [42] and its variants achieve impressive performance and have been applied to perform classification and retrieval tasks. In image analysis, an image is described as a collection of local features from a given vocabulary, resulting in a representation referred to as a bag of features [43,44]. In shape analysis, such methods have been introduced to describe visual words of 3D shape [8,14,45,46,47]. In the literature [48], a hybrid feature descriptor was encoded using codebook for automatic recognition of human interaction. In this work, the Bag-of-Words methodology was applied to construct the SiHKS-BoW descriptor. The details of this mechanism are presented in the following paragraphs.

#### 4.2.1. Evaluating the Si-HKS

**Figure 5:**
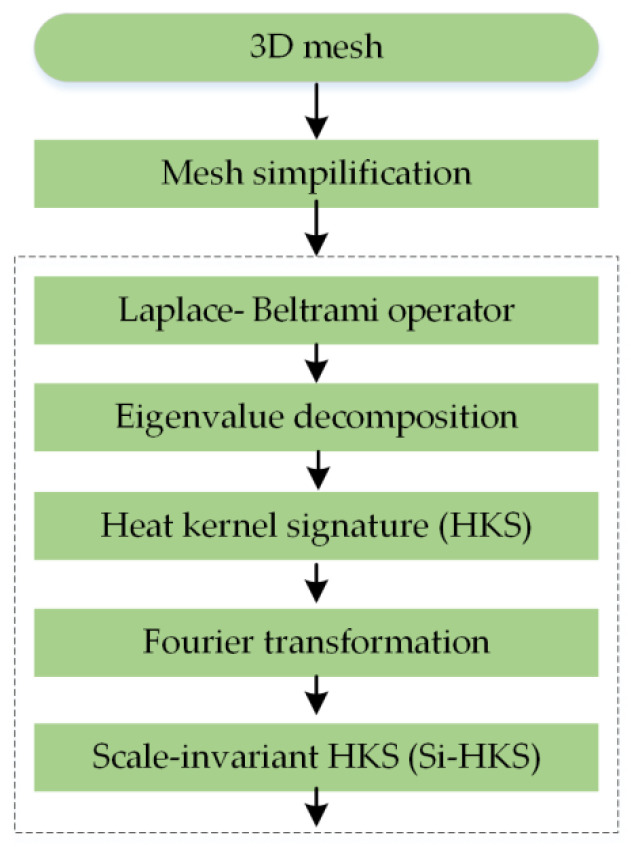
A work-flow for evaluating the Si-HKS.

The Bag-of-Words model represents a 3D shape as a collection of visual words. Figure 5 shows a scheme for evaluating the scale-invariant HKS descriptor. First, the 3D model is simplified with approximately 5000 vertices. Secondly, the Laplace–Beltrami operator of the vertex is calculated and then decomposed to obtain the corresponding eigenvalues and eigenvectors. Then, the first N eigenvalues and their corresponding eigenvectors are selected and substituted into the heat kernel equation to obtain the HKS descriptor. Finally, discrete-time Fourier-transform and phase-amplitude are used to eliminate the scaling instability of the heat kernel signature; thus, the Si-HKS descriptor is obtained.

#### 4.2.2. Visual Codebook Generation

We used the Si-HKS values of densely distributed vertices as elements to construct “geometric words”. To obtain k patterns of all vertices and create the vocabularies, we applied the standard k-means clustering algorithm that was suggested and used in the literature for similar tasks. Then, similar values of Si-HKS were clustered together and assigned the same visual words. As the number of the clusters (K) is much smaller than the number of the vertices (N), the time cost of the similarity calculation was greatly decreased. 

#### 4.2.3. Feature Quantization and Score Calculation

Once we have the codebook, every Si-HKS of a vertex takes a word assigned to its cluster centroid. Si-HKS is represented as a collection of visual “words”, and each 3D shape is converted to a bag of words. Counting the frequency of the visual words in the codebook occurrence, a global feature called “SiHKS-BoW” was constructed.

### 4.3. Classifier

FCM (fuzzy c-means) [49] is one of the best-known clustering algorithms for data mining. Clustering is a process for grouping a set of data into classes so that the data within a cluster have high similarity but are very dissimilar if the data are in different clusters. For traditional FCM, the performance has been limited to the Euclidean distance. We previously proposed an improved FCM algorithm named MKDSIF-FCM [41]. Our experimental results verified the effectiveness and generality of the MKDSIF-FCM algorithm on a publicly available dataset and 3D skull dataset.

The MKDSIF-FCM algorithm puts forward the concept of a distance weighting coefficient with an influence factor (IF) and incorporates the advantage of multiple kernel learning.

Assume X = {x_1_, x_2_, …, x_n_} is a set of m-dimensional samples, where x_j_ = {x_j1_, x_j2_, …, x_jm_} represents the jth sample for j = 1, 2, …, n. The ith cluster is expected to have the center vector v_i_ = {v_i1_, v_i2_, …, v_im_} (1 ≤ i ≤ c), where an integer c (2 ≤ c ≤ n) is the number of clusters.

U∈R_c×n_ is a c × n matrix of fuzzy partition for given x_k_ = {x_k1_, x_k2_, …, x_km_} (k = 1, 2, …, n), where u_ik_∈U is a membership function value from x_k_ to v_i,_ and u_ik_ is subject to the following conditions:

(11) $$\sum_{i=1}^{c}u_{\mathrm{ik}}=1,\forall k$$

(12) $$0\leq u_{\mathrm{ik}}\leq 1,\forall k,i$$

The iterative optimization is used to approximate the minima of an objective function J_S_. In minimizing J_S_, the basic steps are performed in the following procedures:

--Step 1. Given a value of parameters c and let s = 2.

--Step 2. The matrix U of fuzzy partition is initialized by generating c×n random numbers in the interval [0, 1].

--Step 3. For t = 0, 1, 2, …, FCM algorithm is used to calculate v_i_ (i = 1, 2, …, c) by using U as follows:

(13) $$v_{i}=\sum_{k=1}^{n}{\left(u_{\mathrm{ik}}\right)}^{S}x_{k}/\sum_{k=1}^{n}{\left(u_{\mathrm{ik}}\right)}^{S}$$

--Step 4. The w_ik_ is calculated according to (14) and (15),

(14) $$w_{i}=\sum_{k=1}^{n}u_{\mathrm{ik}}\;k=0,1,2,\cdots ,n$$

(15) $$w_{\mathrm{ik}}={\left(w_{i}/u_{\mathrm{ik}}\right)}^{.\wedge \beta }$$

--Step 5. The objective function J_S_ is computed by using (16),

(16) $$J_{s}\left(U,V\right)=\sum_{i=1}^{c}\sum_{k=1}^{n}{\left(u_{\mathrm{ik}}\right)}^{s}{\left\|w_{\mathrm{ik}}\left(K^{*}\left(x_{k}{,x}_{k}\right)+K^{*}\left(v_{i}{,v}_{i}\right)-2K^{*}\left(x_{k}{,v}_{i}\right)\right)\right\|}^{2}$$

--Step 6. The fuzzy partition matrix U and the cluster centers V are updated by minimizing objective function J_S_. The of u_ik_ and v_i_ is calculated according to (17) and (18), respectively.

(17) $$u_{\mathrm{ik}}=\frac{{\left({1-K}^{*}\left(x_{k}{,v}_{i}\right)\right)}^{\frac{-1}{s-1}}}{\sum_{j=1}^{c}{\left({1-K}^{*}\left(x_{k}{,v}_{j}\right)\right)}^{\frac{-1}{s-1}}}$$

(18) $$v_{i}=\frac{\sum_{k=1}^{n}u_{\mathrm{ik}}^{s}K^{*}\left(x_{k}{,v}_{i}\right)x_{k}}{\sum_{k=1}^{n}u_{\mathrm{ik}}^{s}K^{*}\left(x_{k}{,v}_{i}\right)}$$

-- Step 7. The process is stopped if the following condition holds:

(19) $$\left|J_{S}\left(t+1\right){-J}_{S}\left(t\right)\right|<\varepsilon$$

where it converges or the difference between two adjacent computed values of objective functions J_S_ is less than the given threshold ε.

Otherwise, go to step 4.

## 5. Experiment Results and Analysis

### 5.1. Experiment Environment and Testing Dataset

The proposed approach was implemented on an Intel® Core™ i9-9900k CPU @ 3.60 GHz desktop computer with 64 GB RAM and 2 GeForce RTX 2070 GPU running MS Windows 10. The experimental environment was based on MATLAB R2019b, and all 3D models were obtained by an Artec3D Scanner. To verify the effectiveness and show the potential application of our approach, we conducted experiments on the Tang tri-color Hu dataset, which contained 41 samples. The dataset consists of three classes: people, animals, and others. In our experiments, the raw data was simplified as a model with about 5000 vertices and 10,000 faces, as shown in Table A1. In our experimental setup, we closely followed the original works theoretically, and we selected the optimal parameters to yield the best performance on our dataset. For the classification rate and running time presented in this work, the experiments were repeated 50 times, and the average results were obtained for comparison.

### 5.2. Evaluation of Si-HKS Descriptors

In our experiments, 100 eigenvalues and eigenvectors were computed and the heat kernel value at vertex x was computed in the logarithmic scale over the time (t = α^τ^).

#### 5.2.1. Parameter Setting

To construct the HKS descriptor, we used a logarithmic scale-space with base α = 2 and τ ranging from 1 to 30 with increments of 0.5. The heat kernel value of each vertex was a (2 × τ − 1) × 1 dimensional tensor, and a (2 × τ − 1) × n dimensional shape descriptor was obtained by combining the heat kernel values of all the vertices from the 3D models, where n and τ are the number of vertices and the time scale.

To construct the Si-HKS descriptor, the amplitude of the Fourier-transform (FT) was used to achieve scale invariance. Most of the signal information is usually contained in the low-frequency components of the Fourier-transform. In our experiments, the Si-HKS descriptor was sampled at a small number of low frequencies. We set the frequency f ranging from 1 to 40 with increments of 1, and, thus, the first 40 discrete lowest frequencies were used to construct the Si-HKS descriptor.

#### 5.2.2. Performance Evaluation

**Figure 6:**
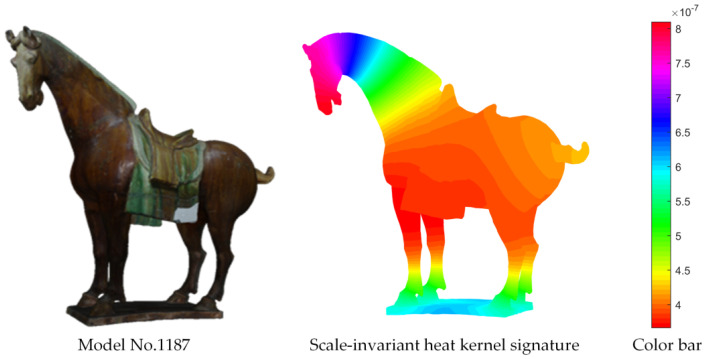
A 3D shape and its heat kernel signature. Left, the 3D Tang tri-color model. Middle, the visual effect of the heat kernel descriptor. Right, the map between color and the value of the heat kernel.

For the chosen 3D cultural relic models, their heat kernel values of all vertices were calculated and visualized. As sketched in Figure 6, the resulting heat kernel distributions were colored according to the values of K_t_ (x, x) at time scale τ = 30, where different colors represent different heat kernel values. 

**Figure 7:**
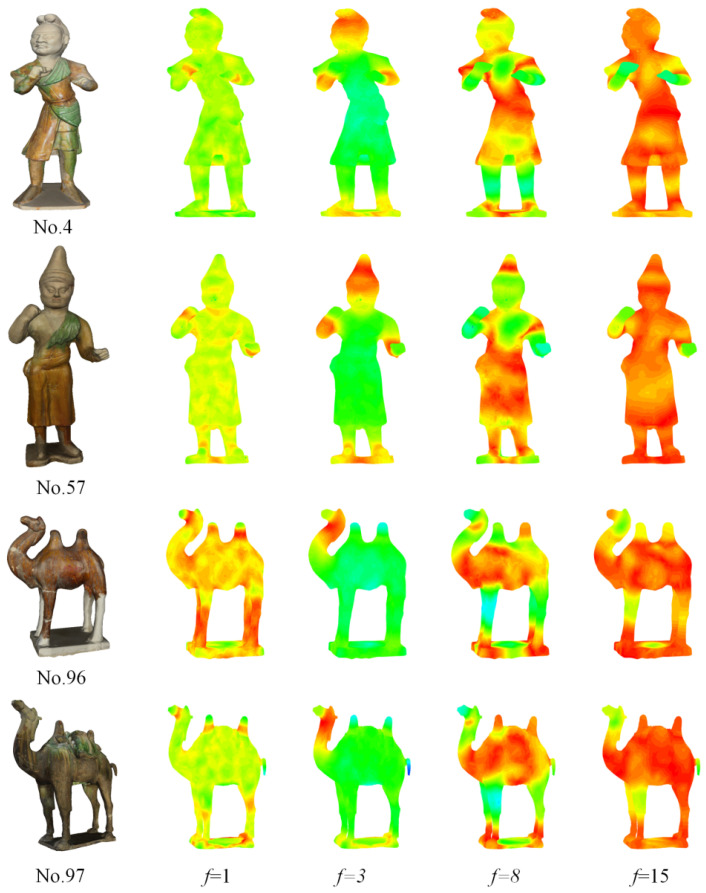
Visual representation of Si-HKS at different frequencies.

We randomly selected f = 1, f = 3, f = 8, and f = 15 from 40 frequencies and mapped the Si-HKS onto 3D cultural relic models (No.4, No.57, No.71, and No.80). As shown in Figure 7, we can see the 3D Tang tri-color models belonging to the same class tended to have similar heat distributions and contained similar visual information. Thus, using Si-HKS descriptors, we can evaluate the similarity between 3D shapes.

**Figure 8:**
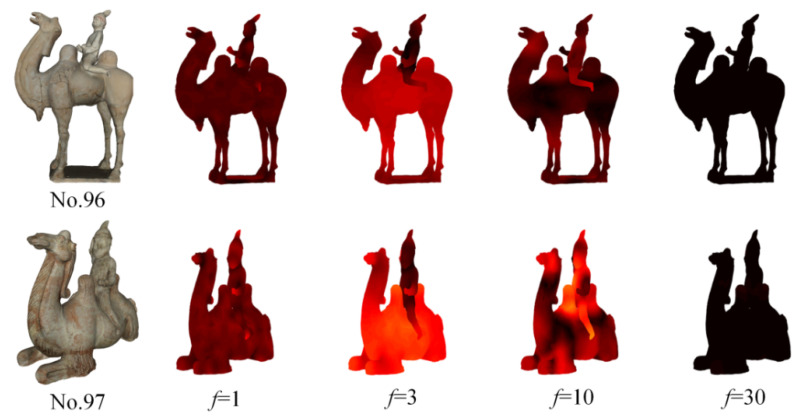
Visual representation of Si-HKS for model No.96 and No.97.

In classification and retrieval tasks, deformations on the body due to movement make recognizing articulated shapes, such as humans or animals, very challenging. The Si-HKS is invariant to isometric deformations. Figure 8 shows the visual representations of Si-HKS for two camels with different poses. Clearly, the heat distributions of models No.96 and No.97 are very similar at different frequencies f.

### 5.3. Evaluation of Mesh Simplification

When scanning the cultural relics by 3D scanner, the obtained 3D data is typically large, and thus leads to high computational complexity and a long computational time. It is often necessary to simplify the original data to an appropriate size. 

As shown in Table 1, when the number of vertices of the model are changed from 670,070 to 5050, the time taken to compute the Si-HKS descriptor is reduced from 2 minutes to less than 1 second, which should be reasonable. It is therefore necessary to simplify the model before feature extraction.

**Figure 9:**
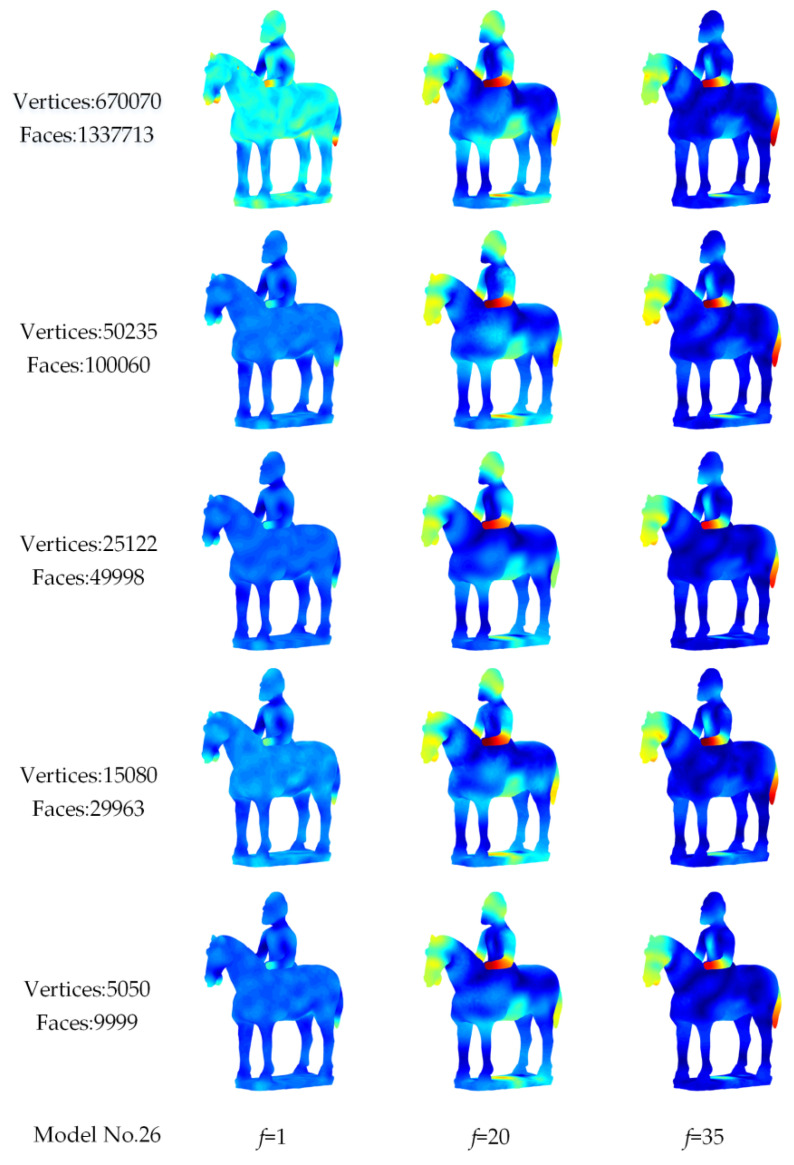
Visual representation of Si-HKS for model No.26 and its simplified versions.

Figure 9 shows the visual representations of Si-HKS for model No.26 and its simplified versions. When the numbers of vertices were reduced from 670,070 to 5050, the heat distribution of Si-HKS on the surface of the simplified model was almost unchanged at different frequencies f compared with the original model. This indicates that the characteristic description ability of the Si-HKS will not be weakened if the model is simplified to an appropriate size.

### 5.4. Evluation of SiHKS-BoW Descriptor

During the construction of the SiHKS-BoW descriptor, the selection of the size of the codebook (K) is very important and can impact the final classification accuracy.

**Figure 10:**
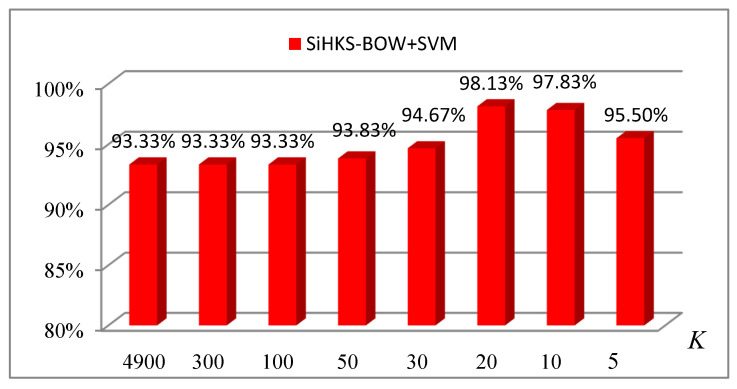
Illustration of the classification accuracy of different codebook sizes (K) in constructing the SiHKS-BOW descriptor.

To obtain the optimal codebook size (K), the SiHKS-BoW was evaluated using the most commonly used classifier, SVM. The evaluation protocol splits the 41 datasets into 26 training samples and 15 test samples. We can see from Figure 10 that a too large or too small value of K may cause the descriptors to be less discriminating and the accuracy to be decreased. In our experiments, the selection of K was mainly by heuristics. The best result up to 98.13% for the SiHKS-BoW descriptor was obtained when the value of K was 20. 

**Figure 11:**
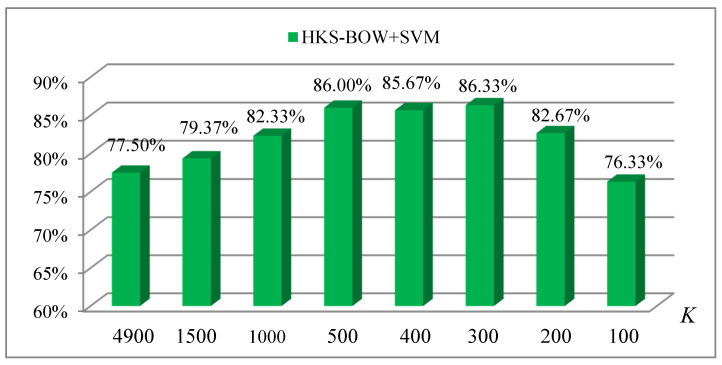
Illustration of the classification accuracy of different codebook sizes (K) in constructing the HKS-BOW descriptor.

In Figure 11, when the value of K was 300, we obtained the highest classification accuracy of 86.33% using the HKS-BoW descriptor. Clearly, the classification accuracy obtained with SiHKS-BoW was 12% higher than that with HKS-BoW. This also implies that the models in our dataset may not be consistent in scale. The scale invariance of the Si-HKS descriptor makes it outstanding in our classification task, which is why we chose the Si-HKS descriptor in our work.

### 5.5. Classifiers Selection

To verify the classification performance of MKDSIF-FCM on the cultural relic dataset, we compared the results using several popular classification methods, including decision tree, BP neural network, SVM, H-ELM [50], and MKDSIF-FCM.

Here, we first focused on a detailed analysis of the parameters of the MKDSIF-FCM algorithm. There are six parameters (s, p_1_, p_2_, σ_1_, σ_2_, and β) that must be set in the MKDSIF-FCM algorithm, where s represents the fuzziness index, p_1_ and p_2_ represent the probability, σ_1_ and σ_2_ represent the parameters of the Gaussian kernel function, and β represents the influence factor (IF). 

The parameters in MKDSIF-FCM were set as s = 2, β = −0.2, p_1_ = 0.7, p_2_ = 0.3, σ_1_ = 30, and σ_2_ = 120.

**Figure 12:**
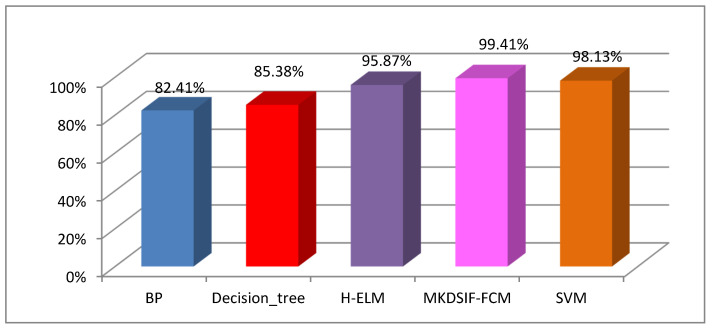
Comparative analysis of the classification accuracies for several popular classifiers on the cultural relic dataset.

The experimental results of Figure 12 suggest that MKDSIF-FCM produced fairly high accuracy at 99.41%, and this was superior to the other classifiers we tested. Both H-ELM and SVM did a good job with a high accuracy over 95%. The classification accuracies of the BP neural network and decision tree were no more than 90%.

Table 2 shows that the longest classification time occurring using BP neural networks, and the shortest was with SVM. As for H-ELM and MKDSIF-FCM, the running time was at a similar level. The decision tree’s running time was slightly longer than H-ELM’s.

As can be seen from the above experimental results, both SVM and MKDSIF-FCM showed excellent classification performance in terms of the classification accuracy and running time. SVM is one of the most popular classifiers that succeeds in object recognition and classification. However, it is a supervised learning method, which requires labelled samples in advance.

### 5.6. Performance Analysis of the Proposed Approach 

**Figure 13:**
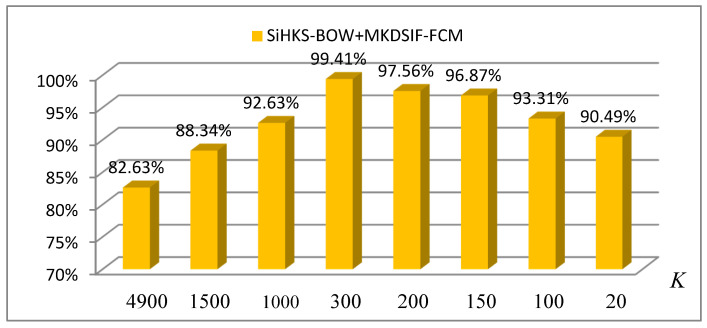
The influence of the codebook size (K) on the classification accuracy.

In general, there is always at least one optimal K that can obtain the highest accuracy when using a Bag-of-Words representation. As shown in Figure 13, when the codebook size (K) is 300, the best average accuracy was obtained using the MKDSIF-FCM algorithm, up to 99.41%. 

#### 5.6.1. Time Cost

The time cost of the proposed approach in each running stage is listed in Table 3. In three stages, the time needed to calculate the Si-HKS descriptor was the longest, up to 46 s. This also shows the necessity of simplifying the model before feature extraction. The time it takes to classify occupies a very small period of time in the whole process of the classification task. The time to construct the SiHKS-BoW descriptor is related to the value of parameter K; the greater the value of K, the longer the time. When the value of K was reduced from 4900 to 20, and the time cost of constructing of SiHKS-BoW was reduced by about 200 s, this had a large impact on the overall running time in the classification task.

In our classification task, when the value of K was 300, we obtained the best classification with 99.41% and the total running time of 60.8793 s that meets the requirements of classification tasks in many real-time scenarios.

#### 5.6.2. Stability Analysis

**Figure 14:**
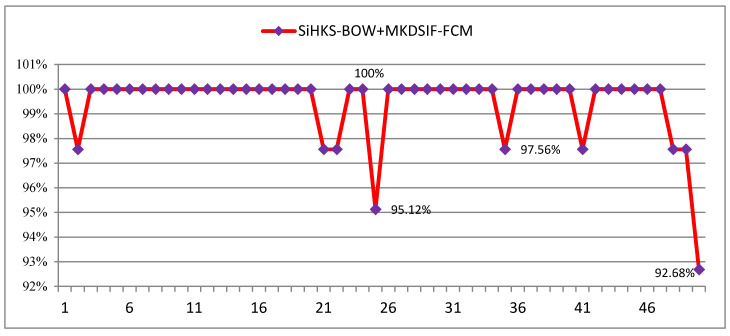
Stability analysis for the proposed approach.

When conducting the classification task, we hoped that the results could be reproduced. It is very important that the method is stable. As shown in the line chart in Figure 14, the stability of the proposed method in detail was further analyzed. The experimental procedure was repeated 50 times, and the difference between maximum and minimum accuracy was around 7%. In 50 experiments, the classification accuracies reached 100% over 41 times. The minimum accuracy was 92.68%. The combination of Si-HKS-BoW + MKDSIF-FCM presented stable performance on our cultural heritage dataset.

## 6. Discussion

The experimental results demonstrated that the Si-HKS descriptor could achieve high performance on our dataset, and its ability to handle data under isometric deformation and scale change make it ideal for our cultural relic classification task.

Extracting the Si-HKS descriptor from a small simplified model is beneficial in improving the computational efficiency. As shown in Figure 9 and Table 1, we can see the time needed to compute the Si-HKS descriptor is greatly reduced after the model simplification, but the discriminative power of descriptors did not change drastically.

Although we simplified the model before feature extraction, the dimensions of the extracted Si-HKS features are still as high as around 5000. A high dimension not only brings a high computational cost, but is also harmful to the classification accuracy. Therefore, it is necessary to further find densities in the feature space and to construct low-dimensional features with good descriptive ability. The size K of the codebook denotes the number of the clustering center. In general, the value of K is much smaller than the number of vertices, and the value of the Si-HKS of each vertex is allocated to each cluster center to achieve the purpose of feature dimension reduction.

There is also a very important objective reason why we constructed the SiHKS-BoW descriptor using the Bag-of-Words methodology. The existing model simplification methods cannot make the number of vertices of the simplified model be the same, which leads to inconsistency of the dimension of the HKS of each model, and also provides trouble for the classification of the later. Therefore, we needed to transform the Si-HKS into a tensor of a uniform size to complete the classification task.

The experimental results fully indicate that compared with the Si-HKS descriptor, a low-dimensional SiHKS-BoW has a considerable benefit to the classification accuracy and the over running time. 

We proposed a stable and efficient unsupervised learning algorithm that we previously abbreviated as MKDSIF-FCM [44] to complete the classification task for a cultural relic dataset. We compared it with several supervised classification methods, including a decision tree, BP neural networks, H-ELM, and SVM. The experimental results showed that MKDSIF-FCM obtained best classification performance for the classification accuracy and time consumption at 99.41% and 0.0024 s, respectively. As a result of the fairly high accuracy, small time cost, and the advantage of unsupervised learning, we have reason to believe that MKDSIF-FCM is the most suitable classifier for cultural relic data in which category labels of the samples are absent or when the sample categories are unbalanced. Our experimental results also indicated that the SiHKS-BoW descriptors we constructed were very accurate and effective in our classification task.

In our experiments, the acquisition of the optimal K was mainly by heuristics. We experimented with all possible values of K rather than choosing a specific value because different K can be useful or harmful depending on the features extracted and the classifier used. How to determine the optimal K automatically with an effective method will be a research topic to investigate in the future.

In the MKDSIF-FCM algorithm, the determination of a set of optimal parameters also depended on heuristics. We will perform more extensive studies on this in the near future and attempt to develop other unsupervised classification methods.

## 7. Conclusions

In this paper, we proposed an efficient classification approach for the Tang tri-color Hu terracotta figures, which was composed of four main phases: (i) data gathering and preprocessing, (ii) estimation of the Si-HKS descriptor, (iii) construction of a new low-dimensional feature using the Bag-of-Words methodology, and (iv) classification. 

The proposed method produced a high accuracy, low time complexity, and stable performance for cultural relic classification while maintaining the advantages of unsupervised learning. We believe that the approach described here is noteworthy for researchers who are attempting (or are considering attempting) to engage in cultural relic classification by means of unsupervised learning methods.

## Figures and Tables

**Table 1:** Comparison of feature extraction times for model No.26 and its simplified versions

NO.	Vertices	Faces	Feature Extraction Time of Scale-Invariant Heat Kernel Signature (Si-HKS) (s)
26	670,070	1,337,713	119.8930
26	50,235	100,060	6.7037
26	25,122	49,998	3.2612
26	15,080	29,963	2.1102
26	5050	9999	0.8156

**Table 2:** Comparative analysis of the time cost for several popular classifiers.

Classifier	BP	Decision Tree	H-ELM	MKDSIF-FCM	SVM
Time(s)	0.6734	0.0133	0.0044	0.0025	0.0022

**Table 3:** Comparative analysis of the time cost of the proposed approach.

Codebook Size (K)	The Time of Calculating Si-HKS (s)	The Time of Constructing SiHKS-BoW (s)	Classification Time (s)	Total Time (s)
20	46.0866	3.3311	0.0022	49.4199
300	46.0866	14.7903	0.0024	60.8793
4900	46.0866	200.5000	0.0023	246.5889

## Appendix A

**Table A1:** The parameters of 3D meshes before and after simplification.

NO.	Raw Data		Simplified Data	
	Vertices	Faces	Vertices	Faces
4	178,814	357,628	4999	9998
7	173,127	346,262	4996	10,000
9	73,994	149,992	4998	10,000
14	743,179	1,485,261	5021	10,000
18	257,234	514,464	5002	10,000
22	709,181	1,416,877	5029	9999
26	670,070	1,337,713	5050	9999
30	1,110,784	2,219,104	5040	9999
36	937,615	1,872,948	4996	10,000
46	147,225	196,978	5016	10,000
55	755,609	1,508,557	5060	10,001
57	128,000	256,000	5000	10,000
58	484,738	969,476	5000	10,000
59	152,346	304,688	5002	10,000
61	295,064	590,128	5000	10,000
65	1,162,107	2,324,182	5009	10,000
66	969,216	1,938,432	5000	10,000
67	343,258	684,106	5009	10,000
70	278,686	557,379	4999	9998
71	296,065	592,137	4996	9999
73	322,850	645,043	5026	10,000
76	412,473	823,926	5049	10,000
77	336,020	670,892	5058	10,000
80	1,084,148	2,164,464	5036	9996
81	700,918	1,400,536	5026	10,000
83	560,879	1,120,130	5035	9999
84	931,203	1,859,687	5049	10,000
85	193,204	386,408	4999	9998
86	471,922	943,844	5000	10,000
87	571,136	1,142,280	4996	10,000
88	272,834	545,676	4996	10,000
89	295,488	590,973	5001	10,000
90	290,325	580,653	4998	9999
91	287,436	574,876	4998	10,000
92	287,700	575,383	5008	9999
93	282,452	564,873	5516	10,999
94	1,558,870	3,117,738	5001	10,000
95	1,630,359	3,260,704	5003	10,000
96	1,010,452	2,020,905	4998	9999
97	1,106,913	2,213,821	5001	9999
1187	1,333,269	2,666,101	5021	9997
